# Significant association between asthma and a lower risk of mortality among COVID-19 patients in Spain: A meta-analysis

**DOI:** 10.5339/qmj.2024.34

**Published:** 2024-07-04

**Authors:** Liqin Shi, Xueya Han, Yadong Wang, Jie Xu, Haiyan Yang

**Affiliations:** 1 Department of Epidemiology, School of Public Health, Zhengzhou University, Zhengzhou, China *Email: yhy@zzu.edu.cn; 2 Department of Toxicology, Henan Center for Disease Control and Prevention, Zhengzhou, China

**Keywords:** Mortality, Spain, asthma, coronavirus disease 2019, severe acute respiratory syndrome coronavirus 2, meta-analysis

## Abstract

**Background:**

Various prevalences of asthma in coronavirus disease 2019 (COVID-19) have been reported in different regions, and the association between asthma and COVID-19 subsequent mortality has been in debate. Thus, this study aimed to investigate whether there was a significant association between asthma and COVID-19 mortality in Spain through a meta-analysis.

**Methods:**

The Preferred Reporting Items for Systematic Reviews and Meta-analyses (PRISMA) guidelines were strictly complied with conducting this study. The pooled odds ratio (OR) with a corresponding 95% confidence interval (CI) was calculated by a random-effects model. The *I*^2^ statistics for heterogeneity, sensitivity analysis for robustness, Begg’s test, and Egger’s test for publication bias, along with subgroup analyses for confounding bias, were also performed to support the foundation of this study.

**Results:**

The meta-analysis revealed that asthma was significantly associated with a lower risk of mortality among COVID-19 patients in Spain with a random-effects model (pooled OR = 0.78, 95% CI = 0.69–0.88, *I*^2^ = 35%). Further subgroup analyses by male proportion and sample size also indicated that a statistically significant negative correlation did exist between asthma and COVID-19 mortality. Robustness and no publication on-bias were evidenced by sensitivity analysis, Egger’s test, and Begg’s test, respectively.

**Conclusion:**

In conclusion, patients with asthma were found to have a lower risk of mortality from COVID-19 in Spain, especially among elderly patients. In addition, asthmatic patients infected with COVID-19 may be at risk of death compared to non-asthmatic patients, which is not a cause for undue concern, thereby reducing the burden of medication.

## Introduction

The novel coronavirus disease 2019 (COVID-19), caused by SARS-CoV-2 (severe acute respiratory syndrome coronavirus 2) infection, has led to over seven million fatalities in infected patients globally as of February 2024, with symptoms of the infection varying widely from asymptomatic to presenting as fatal acute respiratory distress syndrome.^1^ The widespread COVID-19 epidemic poses a serious threat to life and health, especially for older people, males, and people with underlying comorbidities, and the World Health Organization officially announced on May 5, 2023, that COVID-19 no longer constituted a public health emergency of international concern and that surveillance had shifted from emergency response to long-term disease prevention, control, and management.^2,3^ The available evidence suggests that COVID-19 vaccination is the dominant strategy against severe outcomes and mortality of COVID-19,^4^ while asthmatic patients were considered a higher risk group for severe bronchospasm after COVID-19 vaccine injection.^5^ Then asthma was also identified as an independent variable that was significantly associated with low vaccination rates.^6^ Additionally, the Barcelona study showed that asthmatic patients were not susceptible to the SARS-CoV-2 virus, on the contrary, patients with T2 asthma were even immune to the virus to a high degree.^7^

The current debate on COVID-19 has centered on differences between countries, while European countries have adopted different times and methods for self-isolation. As one of the most common non-communicable diseases across Spain, asthma continues to face major diagnostic challenges in adults, such as over- and underdiagnosis.^8^ In Spain, the average prevalence of asthma in adults is 5%, and in children, it is twice as high, while uncontrolled asthma is mostly underdiagnosed by clinicians.^9^ During the global COVID-19 pandemic, there was a decrease in asthma exacerbations and influenza-related illnesses that might have been due to public health measures. Several individual studies also explored the relationship of comorbid asthma with the risk of COVID-19 mortality in Spain, but the conclusions obtained from these articles were inconsistent.^10-12^ Previous meta-analyses have also explored the interaction between asthma and the risk of mortality in patients with COVID-19, but they failed to observe the consistent effect of asthma on COVID-19 mortality in different regions (Appendix Table 1). In fact, geographic differences in the prevalence of asthma or methods of ascertainment may be accounting for these heterogeneous findings.

Therefore, investigating the relationship between asthma and the risk of mortality in COVID-19 patients in a specific country was motivated to be on the agenda. Meanwhile, there has not been a quantitative meta-analysis on this topic in Spain. To sum up the above, a quantitative meta-analysis is called to clarify the relationship of asthma with COVID-19 mortality in Spain to provide guidelines and relieve the burden and severe outcome of COVID-19 for the specific country or region.

## Methods

### Data sources and search strategy

The Preferred Reporting Items for Systematic Reviews and Meta-analyses (PRISMA) guidelines were strictly adhered to in conducting this meta-analysis.^13,14^ PubMed, Elsevier ScienceDirect, Web of Science, Springer Link, Wiley Library, and Cochrane Library were utilized to generally search all potential studies as of September 10, 2023. To expand the search while maintaining relevance to the topic, the following keywords were applied: “SARS-CoV-2” or “severe acute respiratory syndrome coronavirus 2” or “coronavirus disease 2019” or “COVID-19” or “2019 novel coronavirus” or “2019-nCoV” and “asthma” or “asthmatic” and “deceased” or “dead” or “death” or “fatality” or “mortality” or “non-survivor” and “Spain” or “Spanish” to identify studies written in English. The main ending variable was focused on mortality. At the same time, the references in the list of relevant studies were also considered to identify potential branches of the overall research. When publications with data derived from the same period and location were found, only the articles with a larger sample size or a more complete analysis were included in the study.

### Article eligibility criteria

After removing duplicates, two separate investigators evaluated the inclusion eligibility of studies by assessing the titles, abstracts, and full texts. Studies were included in the meta-analysis if the following criteria could be met: (1) all patients were confirmed with COVID-19 infection in Spain; (2) peer-reviewed articles published in English; and (3) articles clearly reported the number of alive and dead with comorbid asthma or not in COVID-19 patients, or the effect size with a 95% confidence interval (CI) regarding the relation between asthma and COVID-19 mortality. Preprints, reviews, case reports, errata, and studies without available data were excluded.

### Data extraction and analysis

The following main information was extracted in order: first author, sample size, the mean (standard deviation) or median (interquartile range) age, proportion of males, available data on the incidence of the alive and the dead among COVID-19 patients or the effect size with 95% CI, and setting from each study. The process was carried out by the two independent investigators. Once divergences happened, the next negotiating situation would come into effect, and the third person made the final decision.

The pooled odds ratio (OR) and 95% CI were calculated by a random-effects model using R software (version 4.2.2). Heterogeneity among individual studies was evaluated using the *I*^2^ statistic.^15^ Sensitivity analysis, omitting each article, was applied to examine the stability of our results. Publication bias was valued through Begg’s rank correlation test and Egger’s linear regression test.^16,17^ The standard of statistical significance was defined as a two-tailed *p* value <0.05.

## Results

### Study selection and study characteristics

**Figure 1.**
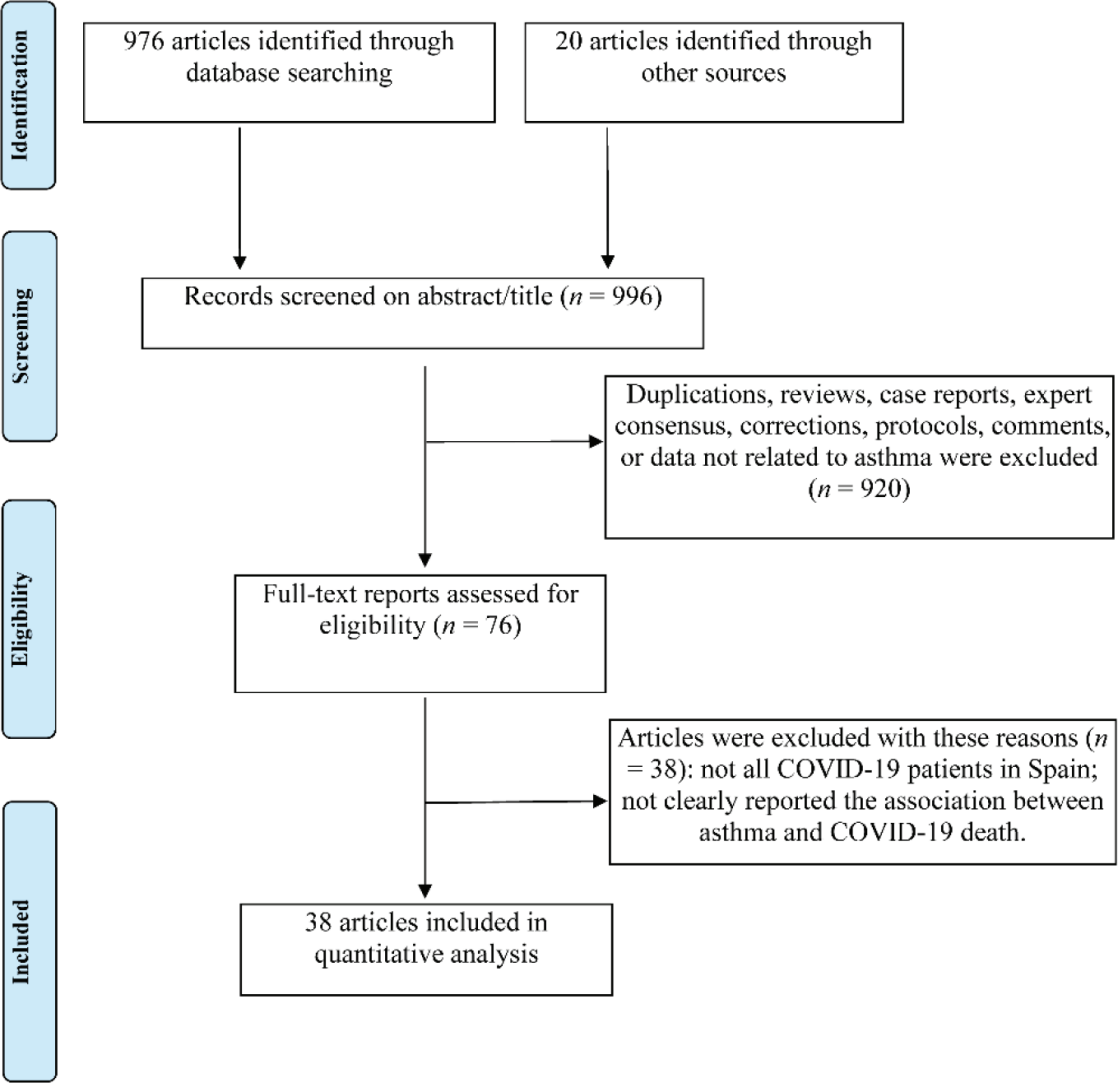
PRISMA flow diagram.

The PRISMA flow diagram shown in Figure 1 depicts our search methodology. The online literature search provided 976 articles from electronic databases. Twenty references of the included studies or reviews were retrieved via hand-searching to obtain as many relevant studies as possible. After screening all abstracts/titles, 920 full-text articles were excluded. A further 38 studies were excluded after a detailed assessment of the number of the alive and the dead with comorbid asthma or the effect size with a 95% CI according to inclusion and exclusion criteria. In total, 38 studies containing 260,351 COVID-19 patients were included in this meta-analysis.^10-12^^,^^18-52^ All studies were related to Spain, with sample sizes ranging from 57 to 86,867 patients based on the clear endpoint of death. Thirty-one studies were based on hospitalized patients, and the remaining seven studies were based on all patients and addressed the relationship between asthma and COVID-19 mortality. The age of the patients included in the eligible studies ranged from 36.9 to 75.9 years. In most studies, more than half of the participants were male. Of interest, the prevalence of asthma in COVID-19 patients varied from 2 to 12.8% among the included studies. The primary characteristics of eligible studies are presented in Table 1.

### Meta-analysis of asthma and mortality in COVID-19 patients

**Figure 2.**
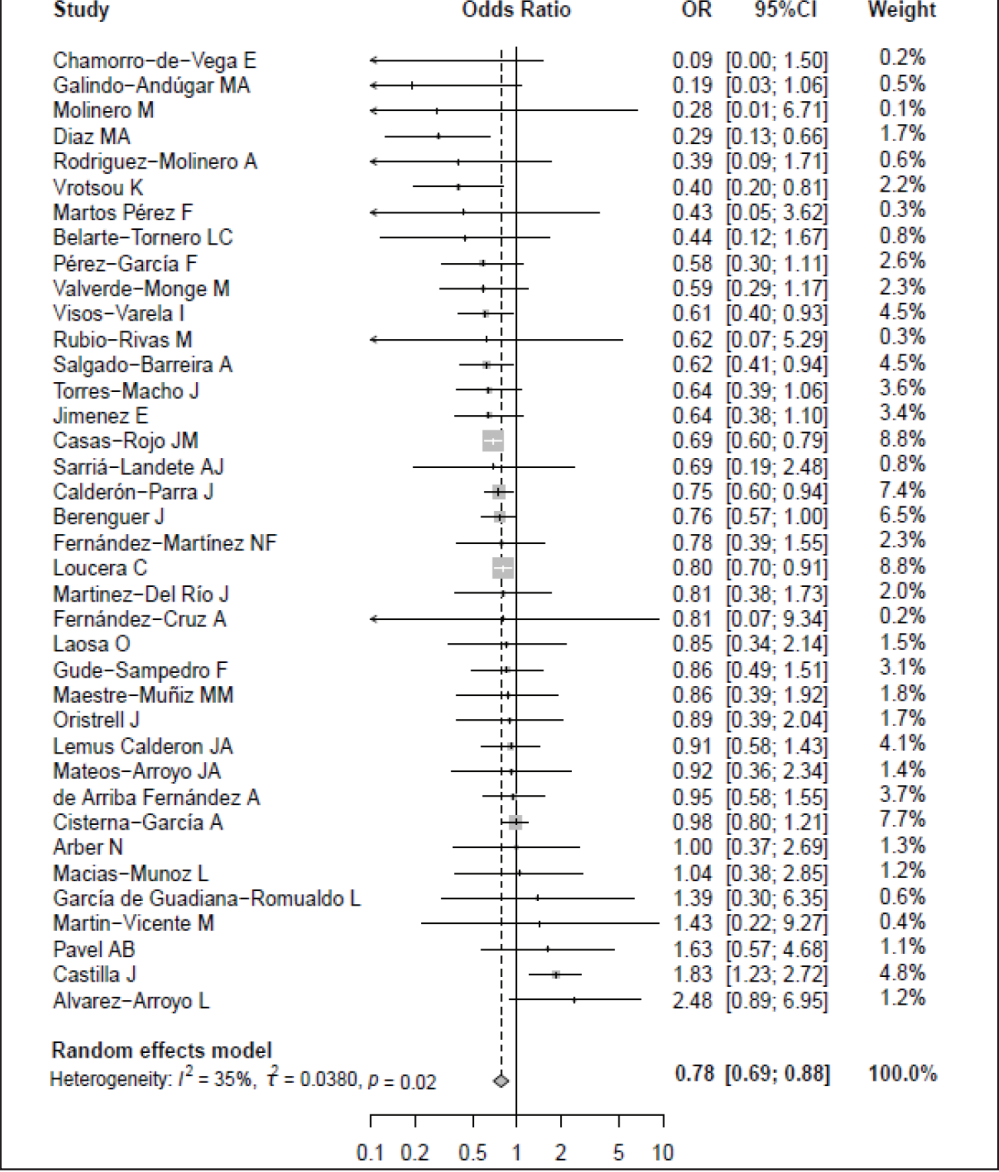
Forest plot for the relationship between asthma and the risk of mortality among coronavirus disease 2019 (COVID-19) patients with pooled odds ratio (OR) and 95% confidence interval (CI).

The meta-analysis revealed that asthma was significantly associated with a lower risk of mortality among COVID-19 patients in Spain with a random-effects model (pooled OR = 0.78, 95% CI = 0.69–0.88, *I*^2^ = 35%, Figure 2). This indicates that asthma in COVID-19 patients reduced the risk of death by 22% as compared to non-asthmatic patients. When the participants were only restricted to hospitalized COVID-19 patients, COVID-19 patients with asthma still tended to be a low-risk group for mortality compared to those without asthma (pooled OR = 0.73, 95% CI = 0.68–0.79) without heterogeneity. Subgroup analyses by male proportion and sample size all yielded consistent results on a significantly negative association between asthma and COVID-19 mortality in the subgroup of male proportion ≥55% (pooled OR = 0.72, 95% CI = 0.65–0.79), male proportion <55% (pooled OR = 0.79, 95% CI = 0.64–0.97), sample size ≥1500 (pooled OR = 0.77, 95% CI = 0.66–0.90), and sample size <1500 (pooled OR = 0.78, 95% CI = 0.63–0.97). Subgroup analysis by age demonstrated that patients with asthma had a reduced risk of COVID-19 mortality, especially for patients aged ≥60 years (pooled OR = 0.73, 95% CI = 0.67–0.79) but not for those aged <60 years (pooled OR = 0.97, 95% CI = 0.70–1.34).

### Sensitivity analysis and publication bias

**Figure 3.**
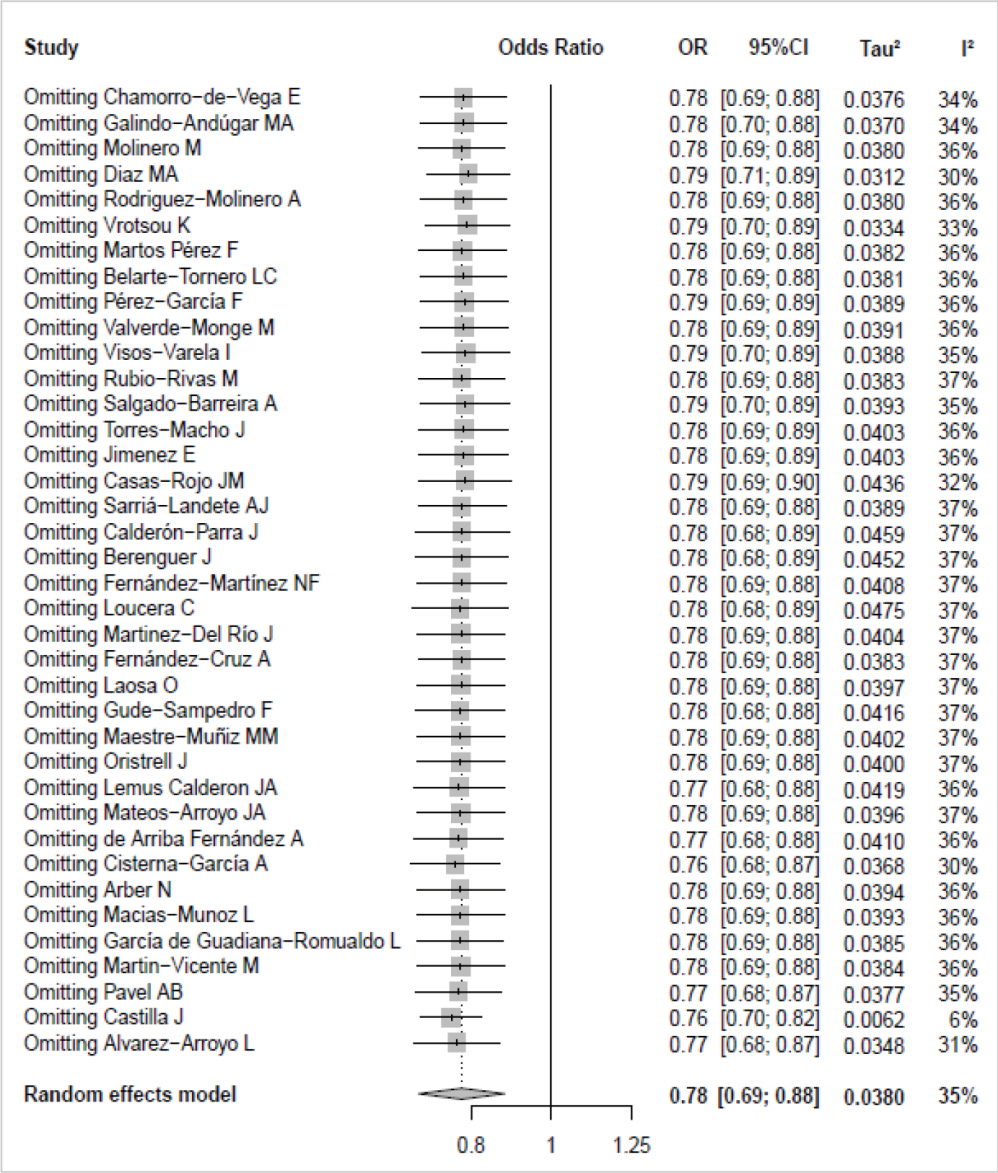
Sensitivity analysis for pooled OR and 95% CI by omitting one study at a time.

**Figure 4.**
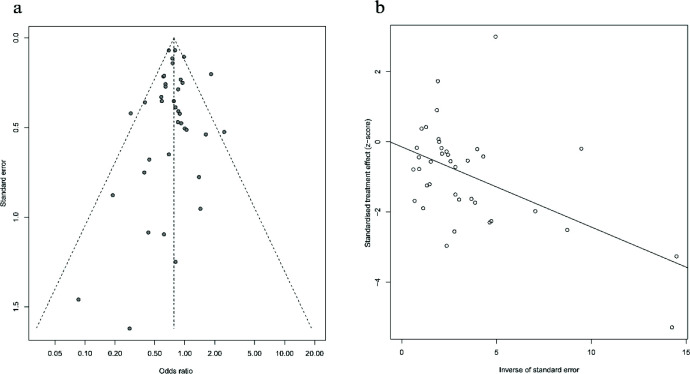
Plots of publication on-bias: (A) based on Begg’s test and (B) based on Egger’s test.

As shown in Figure 3, the forest plot indicated that the pooled OR did not change significantly by the singular exclusion of the included studies, which indicated the robustness of this study. The *p*-values for the Begg test (Figure 4A) and the Egger test (Figure 4B) were 0.870 and 0.642, respectively, and these results did not provide evidence of a significant effect of publication bias.

## Discussion

Understanding the risk variables is critical to selecting those who should be vaccinated first. Until now, sex, age, and specific previous medical histories have all been found to be risk factors for COVID-19 mortality.^53-55^ It is not widely known whether asthma additionally serves as an aggravating factor for worsening COVID-19 outcomes. To our knowledge, this is the first quantitative meta-analysis to investigate the impact of asthma on the risk of COVID-19 mortality in Spain. The data based on 38 studies involving 260,351 COVID-19 patients indicated that asthma was a significant protective factor for COVID-19 death in Spain. Further subgroup analyses by male proportion and sample size also indicated that COVID-19-infected patients with asthma had a lower risk of dying, especially among older patients. The lower heterogeneity of the included studies indicated the reliability of the results and also excluded, to some extent, the interference of the diversity of COVID-19 prevention and control measures in different regions.

Despite the under- and over-diagnosis of asthma in Spain, as mentioned in the introduction, our study suggested that asthma was a protective factor from COVID-19-induced death, especially during the pandemic. This seems like a good surprise for the routine control of asthma. The main focus of asthma management is on the routine control of asthma symptoms, reducing the risk of worsening symptoms and hospitalization, and at the same time minimizing the adverse effects of medications, especially inhaled corticosteroids. Corticosteroids were also used to prevent and control disease progression during the SARS (severe acute respiratory syndrome coronavirus) epidemic and the early stages of the COVID-19 infection. This may partly explain our results and also provide some suggestions for the management of asthma during the pandemic.

The COVID-19 vaccines provide strong protection against severe illness and death.^56,57^ Although it is still possible to get COVID-19 after vaccination, they are more likely to have mild or no symptoms.^58^ Identifying populations contraindicated to vaccination and those at high risk of poor prognosis is the major area of concern beyond COVID-19. The rate of allergic reactions to the COVID-19 mRNA vaccine is higher in patients with a history of high-risk allergies.^59,60^ As we know, allergic asthma is the most common type of asthma and is accompanied by eosinophilic airway inflammation, massive mucus production, airway hyper-responsiveness, and reversible airway obstruction. Asthmatic patients have been reported to experience significant bronchospasm within 10–20 minutes after the first dose of the mRNA COVID-19 vaccine.^61^ However, there was sporadic evidence suggested that adverse reactions such as acute bronchospasm were rare in patients with severe asthma after the COVID-19 vaccination.^62^ Additionally, studies involving larger sample sizes have shown that vaccines were effective in preventing hospitalization for COVID-19 and respiratory failure complicating COVID-19 among patients with chronic respiratory diseases (including asthma).^63^ Further research to elucidate the safety of the COVID-19 vaccination for asthmatic populations is needed. A recent study found lower rates of COVID-19 vaccination among young people from families with lower socioeconomic status and with asthma, including those with uncontrolled asthma.^64^ Bossios et al. investigated the experience of COVID-19 vaccination in patients with severe asthma and found that vaccination had little effect on asthma control.^65^ In contrast, another study showed that asthmatic patients treated with biologic therapies such as benralizumab (IL-5 receptor antagonist) or mepolizumab (IL-5 antagonist) had a reduced COVID-19 vaccine response.^66^ Although more than 86% of the Spanish population is currently vaccinated with full doses, only about 2% of Spaniards who are eligible for the COVID-19 vaccine have refused it, including those who could not be vaccinated for medical reasons.^67^ However, there are few available data on COVID-19 vaccination for asthmatic patients in Spain.

On the one hand, the incidence of respiratory disease caused by rhinoviruses and viral loads is higher in asthmatic patients compared to healthy subjects. Once asthma was out of effective control, the severity of virus-induced deterioration worsened dramatically.^68^ Additionally, when asthmatic children presented to the emergency department with COVID-19, they were more likely to develop severe disease than patients without asthma.^69^ Conversely, on the other hand, there was also evidence that asthma did not impact negatively but rather positively on the outcomes of COVID-19.^70,71^ This was consistent with our findings, especially among elderly patients. Although previous studies have shown that old age is a risk factor for COVID-19 infection and asthma, even with a poor prognosis.^55^ However, in our study, a phenomenon worthy of attention is that ≥60-year-old asthmatic patients had a relatively lower risk of death in COVID-19 compared to the <60-year-old group (0.73 vs. 0.97). Despite the fact that the exact reasons for the potentially protective effect of asthma on COVID-19 death in elderly patients in our study are not known, we hypothesize that this may be partly attributable to the following reasons. In part, it is due to the fact that there are nearly 10 million Spanish citizens over the age of 65 years with a high life expectancy and that the vaccination strategy in Spain during the pandemic was age-segmented,^72^ progressively moving from the older to the younger age groups, with a 90% vaccine coverage in the over 60-year-old age group but a lower percentage of vaccinations in younger age groups. The other is partly attributable to the clinically intensive treatment of COVID-19 elderly patients with asthma. Asthma constituted the majority of the disability-adjusted life years index, with the highest incidence rate in the young group.^73^ This suggests a putative mechanism by which asthma may be responsible for death from the COVID-19 infection. The pathophysiology of asthma concentrates on two primary immunologic pathways; both endotypes are usually categorized as airway eosinophilia and type-2-inflammation.^74,75^ Of these, peripheral blood eosinophil counts served as markers to identify endotypes and for asthma treatment, and eosinophils may also promote protective responses against respiratory viruses.^76,77^

In such a situation, possible molecular mechanisms of SARS-CoV-2 infection causing death with asthma seemed to be explained in recent studies. Angiotensin-converting enzyme 2 (ACE2) could act as a cellular receptor for SARS-CoV-2, just like SARS-CoV. Higher ACE2 gene expression was found to be positively associated with COVID-19, both in terms of susceptibility78 and severity of COVID-19.^79^ However, reducing ACE2 expression was found in respiratory allergies and controlled allergen exposures, including allergic sensitization and asthma.^80^ The transmembrane protease serine 2 (TMPRSS2) facilitated virus entry and propagation in host cells by viral cleavage of the viral stinger protein, enabling SARS-CoV-2 to bind efficiently to ACE2.^81-85^ The expression of ACE2 and TMPRSS2 was lower in the respiratory epithelial cells of asthma patients taking inhaled corticosteroids.^86^ Corticosteroids, which are routinely used to treat asthma, affect the expression of the ACE2 or TMPRSS2 gene in sputum cells, and other drugs for asthma might decrease inflammation and enhance antiviral defense.^87,88^ In addition, individuals with asthma were considered to be at higher risk of unfavorable clinical outcomes from COVID-19 in the early phase of the pandemic, which made COVID-19 patients with asthma receive more medical care and preferential treatments. The above explanations might shed some light on the relationship between asthma and the lowered risk of COVID-19-related death. Further evidence focusing on the underlying molecular mechanisms is required to validate the observed association.

Several limitations inevitably existed in this meta-analysis. First, the pooled OR was estimated on the unadjusted effect sizes, which meant the role of confounding factors might be overlooked. Therefore, risk factor-adjusted estimates would be necessary to verify the current findings. Second, as a sort of chronic disease, asthma type, severity, vaccination status, and routine medications might account for the correlation with COVID-19 mortality. However, most of the articles included in this study did not clearly report on the contents mentioned above. These reasons did impose restrictions on the robust generalization of the association, and further study is still needed to elucidate the relationship between COVID-19 and asthma. Third, the participants mainly consisted of adult COVID-19 patients in the present study, and the relationship between asthma and COVID-19 mortality among children in Spain should be focused on in the future when more data are available.

## Conclusion

Patients with asthma were found to have a lower risk of death compared with patients without asthma in Spain, especially among elderly patients. The risk of serious outcomes after a COVID-19 infection may be reduced by maintaining asthma control. In addition, there is no cause for alarm that asthmatic patients infected with COVID-19 may be at risk of death compared to non-asthmatic patients, thereby reducing the burden of medication.

## Authors’ Contributions

HY and YW designed the study. LS, XH, JX, and HY performed literature searches and data extraction. LS, XH, JX, and YW conducted statistical analyses. LS wrote the manuscript. All authors read and approved the final manuscript.

## Acknowledgments

We would like to thank Shuwen Li, Mengke Hu, Ruiying Zhang, Jiahao Ren, Jian Wu, Yang Li, Peihua Zhang, Xuan Liang, Wenwei Xiao, Ying Wang, and Li Shi (all from the Department of Epidemiology, School of Public Health, Zhengzhou University) for their kind help in searching articles, collecting data, and valuable suggestions for data analysis.

## Source of Supporting and Funding

This study was supported by grants from the National Natural Science Foundation of China (No. 81973105), the Key Scientific Research Project of Henan Institution of Higher Education (No. 21A330008), the Joint Construction Project of Henan Medical Science and Technology Research Plan (No. LHGJ20190679), and the Henan Young and Middle-aged Health Science and Technology Innovation Talent Project (No. YXKC2021005). The funders have no role in the data collection, data analysis, preparation of the manuscript, and decision to submission.

## Data Availability Statement

The data used and/or analyzed during the current study are available from the corresponding author on reasonable request.

## Ethical Approval

Not applicable.

## Conflict of Interest Statement

All authors report that they have no potential conflicts of interest regarding this submitted manuscript.

## Figures and Tables

**Table 1.** General information of the eligible studies included in this meta-analysis.

				**Asthama**		**No asthama**				
**Author**	**Sample size**	**Male (%)**	**Age**	**Non-survivor**	**Survivor**	**Non-survivor**	**Survivor**	**Asthma (*n*/%)**	**Setting**	**Refs.**
Vrotsou K	14,179	38.9	53.7 ± 17.4	8	346	757	13,068	354 (2.4%)	All patients	^10^
Gude-Sampedro F	10,454	39.9	58 ± 20	13	275	531	9635	288 (2.8%)	All patients	^12^
Alvarez-Arroyo L	255	54.9	70.0 (52.0–82.1)	6	13	37	199	19 (7.5%)	Hospitalized	^18^
Arber N	448	63.8	65 (54–76)	Effect (95% CI): 1.0 (0.4–2.9)				33 (7.4%)	Hospitalized	^19^
Belarte-Tornero LC	129	48.8	80.3 ± 12.0	3	11	44	71	14 (10.9%)	Hospitalized	^20^
Berenguer J	4035	61	70 (56–80)	69	230	1062	2674	299 (7.4%)	Hospitalized	^21^
Calderón-Parra J	14,973	56.5	69 (56–79)	Effect (95% CI): 0.75 (0.60–0.94)				1097 (7.3%)	Hospitalized	^22^
Casas-Rojo JM	23,983	57.5	69 (56–80)	239	1436	4351	17,957	1675 (7.0%)	Hospitalized	^23^
Castilla J	35,387	48.5	36.9	28	2302	218	32,839	2330 (6.6%)	All patients	^24^
Chamorro-de-Vega E	162	70.4	64 (53–72)	0	10	54	98	10 (6.2%)	Hospitalized	^25^
Cisterna-García A	86,867	47.4	38.9 ± 22.7	97	7403	1044	78,323	7500 (8.6%)	All patients	^26^
de Arriba Fernández A	19,850	47.1	40.7 ± 17.7	18	2530	129	17,173	2548 (12.8%)	All patients	^27^
Diaz MA	2715	53.6	61.3 ± 19.0	6	124	383	2202	130 (4.8%)	Hospitalized	^28^
Fernández-Cruz A	71	62	68.6 ± 13.9	1	2	26	42	3 (4.2%)	Hospitalized	^29^
Fernández-Martínez NF	968	54.8	67 (55–77)	10	59	161	738	69 (7.1%)	Hospitalized	^30^
Galindo-Andugar MA	378	51.9	73.3 ± 14.5	Effect (95% CI): 0.190 (0.034–1.061)				24 (6.3%)	Hospitalized	^31^
García de Guadiana-Romualdo L	359	64.1	59 (47–71)	2	15	30	312	17 (4.7%)	Hospitalized	^32^
Jimenez E	1393	57.5	68.48 ± 18.34	17	95	279	1002	112 (8.0%)	Hospitalized	^33^
Laosa O	373	55.2	66.06 ± 15.33	6	28	68	271	34 (9.1%)	Hospitalized	^34^
Lemus Calderon JA	6310	41	59 ± 19	21	556	229	5504	577 (9.1%)	All patients	^35^
Loucera C	15,953	54.7	68.1	274	1638	2432	11,609	1912 (12.0%)	Hospitalized	^36^
Macias-Munoz L	500	57.4	64 (54–76)	5	20	92	383	25 (5.0%)	Hospitalized	^37^
Maestre-Muñiz MM	444	56.5	71.2 ± 14.6	9	22	133	280	31 (7.0%)	Hospitalized	^38^
Martin-Vicente M	92	65.2	66.0 (50.0–71.5)	1	1	37	53	2 (2.2%)	Hospitalized	^39^
Martinez-Del Río J	921	54.3	75.9 ± 12.0	9	30	239	643	39 (4.2%)	Hospitalized	^40^
Martos Pérez F	96	66	63 ± 17	1	10	16	69	11 (11.5%)	Hospitalized	^41^
Mateos-Arroyo JA	312	54.2	68.72 ± 15.87	6	33	45	228	39 (12.5%)	Hospitalized	^42^
Molinero M	57	80.7	63 (59–70)	0	3	18	36	3 (5.3%)	Hospitalized	^43^
Oristrell J	5885	NR	NR	Effect (95% CI): 0.89 (0.39–2.05)				NR	All patients	^44^
Pavel AB	288	60.8	64 ± 15	5	16	43	224	21 (7.3%)	Hospitalized	^45^
Pérez-García F	1200	59.5	67.2 ± 18.5	11	85	201	903	96 (8.0%)	Hospitalized	^46^
Rodriguez-Molinero A	418	56.9	65.4 ± 16.6	2	21	77	318	23 (5.5%)	Hospitalized	^47^
Rubio-Rivas M	186	69.4	64.3 ± 13.0	1	6	38	141	7 (3.8%)	Hospitalized	^48^
Salgado-Barreira A	3060	50.7	74 (59–84)	26	259	387	2388	285 (9.3%)	Hospitalized	^49^
Sarriá-Landete AJ	322	50.9	68.4 ± 15.2	3	14	72	233	17 (5.3%)	Hospitalized	^50^
Torres-Macho J	1968	56.1	67	18	138	307	1505	156 (7.9%)	Hospitalized	^51^
Valverde-Monge M	2539	50.2	62.66 ± 19.05	9	104	311	2115	113 (4.5%)	Hospitalized	^52^
VisosVarela I	2821	51.6	74 (60–85)	25	242	372	2182	267 (9.5%)	Hospitalized	^53^

The age (years) was presented as mean ± standard deviation or median (interquartile range, IQR).

CI: confidence interval, NR: not clearly reported.

## Appendix

**Appendix Table 1.** The summary of prevalence of asthma among COVID-19 patients and the risk of COVID-19 mortality in the previous meta-analyses.

**Study**	**Cases of COVID-19**	**Prevalence% (95%CI)**	**Pooled effect (95%CI)**
Terry PD^1^	878,239	7.1% (5.6–8.8%)	RR 0.89 (0.77–1.02)
USA		11% (9.8–12.3%)	
Mexico		2.9% (2.8–3.1%)	
Europe		7.6% (6.0–9.4%)	
China		1.9% (0.4–4.4%)	
South Korea		5.4% (2.3–9.6%)	
			
Sunjaya AP^2^	1,471,643	8.80% (6.87–9.30%)	RR 0.94 (0.76–1.17)
Asia			RR 2.01 (1.19–3.39)
Europe			RR 0.85 (0.68–1.05)
North America			RR 0.79 (0.58–1.06)
South America			RR 0.72 (0.47–1.12)
			
Hussein MH^3^	107,983	11.2% (9.1–13.3%)	RR 0.80 (0.65–0.97)
USA		12.8% (9.3–16.3%)	
Europe		12.7% (2.7–22.8%)	
Mexico		2.9% (2.8–3.0%)	
Liu S^4^	410,382	1.1%–16.9%	RR 0.65 (0.43–0.98)
China mainland		1.5%	
South Korea		16.9%	
Japan		5.7%	
France		6.6%	
United Kingdom		14.5%	
Portugal		1.4%	
Italy		1.1%	
Spain		4.9%	
Mexico		2.8%	
United States		10.2%	
Brazil		5.7%	
			
Shi L^5^	403,392	8.3% (7.6–9.0%)	RR 0.88 (0.73–1.05)
Asia		3.3% (1.9–4.6%)	
The Americas		7.0% (5.0–9.0%)	
Europe		9.4% (6.2–12.5%)	
The Middle East		2.0% (1.8–2.1%)	
			
Sitek AN^6^	92,275		OR 0.73 (0.38–0.40)
			
Soeroto AY^7^	6046		OR 0.81 (0.56–1.15)
			
Wang Y^8^	17,694		OR 1.03 (0.55–1.93)
			
Hou H^9^	2,457,205		OR 0.87 (0.83–0.92) HR 0.90 (0.83–0.97)
Europe			OR 0.86 (0.73–1.01)
North America			OR 0.87 (0.82–0.92)
Asia			OR 1.13 (0.92–1.38)
South America			OR 0.81 (0.77–0.86)
Africa			OR 1.52 (0.41–5.60)
			
Pardhan S^10^	1,678,992		OR 0.83 (0.71–0.96) HR 0.93 (0.87–1.00)
			
Wu X^11^	1,169,441		OR 0.95 (0.78–1.15)
			
Wang Y^12^	8895		OR 0.96 (0.70–1.30)
			
Reyes FM^13^	10,136		OR 0.87 (0.68–1.10)

CI: confidence interval, HR: hazard ratio, OR: odd ratio, RR: risk ratio, USA: the United States of America.
